# A study on sublabial transsphenoidal treatment of pituitary tumor under microscope with aid of endoscope

**DOI:** 10.1186/s41016-018-0130-y

**Published:** 2018-09-03

**Authors:** Yunchol Pak, Xuejun Yang, Yongdok Kim, Chanhong Jong, Haksong Kim, Namhyok Lee, Songgun Kim, Yongchol Kim

**Affiliations:** 1 Department of Neurosurgery, Pyongyang Medical School Hospital, Kim Il Sung General University, Pyongyang, Democratic People’s Republic of Korea; 2 Department of Neurosurgery, General Hospital, Tianjin Medical University, Tianjin, China

**Keywords:** STT approach, Endoscopic, Sellar, Pituitary surgery, Complication

## Abstract

**Background:**

A pituitary tumor is a tumor with a high incidence among brain tumors. Microscopic traditional STT approach with aid of endoscope has a high therapeutic effect and few complications.

**Methods:**

The authors reviewed 146 patients who underwent STT resection of sellar masses between January 1, 2006, and December 31, 2013. From this group, 40 patients who underwent microscopic STT surgery with aid of endoscope were included in the study. In this series, there were many nonfunctional and Growth Hormone(GH) secreting adenoma. In the study group, tumor size ranged from 60 to 79 mm in 3 cases. Pathophysiological studies, tumor sizes, clinical outcomes, extent of resection, visual function and complication were estimated.

**Results:**

In our study, gross-total resection(GTR) was achieved in 33 patients (82.5%), subtotal in 3 (7.5%), and partial in 4 (10%). Visual acuity were improved in 82.1%, while 17.9% were unchanged. Visual fields were improved in 81.2%. No deaths occurred in this cohort of patients.

**Conclusions:**

Pituitary adenoma surgery under microscope with aid of endoscope is an effective treatment method, which results in high (> 90%) rates of resection. It is not associated with high rates of major complications and is safe when performed by experienced surgeons.

## Background

Pituitary adenomas comprise about 10–12% of all intracranial tumors [1]. The microscopic STT approach has been recognized as a less-invasive surgery for pituitary tumors [2, 3]. Endoscopic endonasal pituitary tumor surgery provides a more clear and panoramic view and made it possible to distinguish the boundary between the tumor, the cavernous sinus and the arachnoid membrane [4–10]. However, postoperative CSF leak and some infectious complications has not disappeared and has become a frequent complication [11–17]. Nasal septum bone flap is reported to very effective for restoration of dural defect in sellar area after tumor removal [18–22]. We describe a method of obtaining a bone flap necessary for the sellar floor repairment using the traditional STT approach, and exploring and remove a residual tumor with a endoscope, which simply not visible with microscope [23].

## Method

### Patient materials

**Fig. 1:**
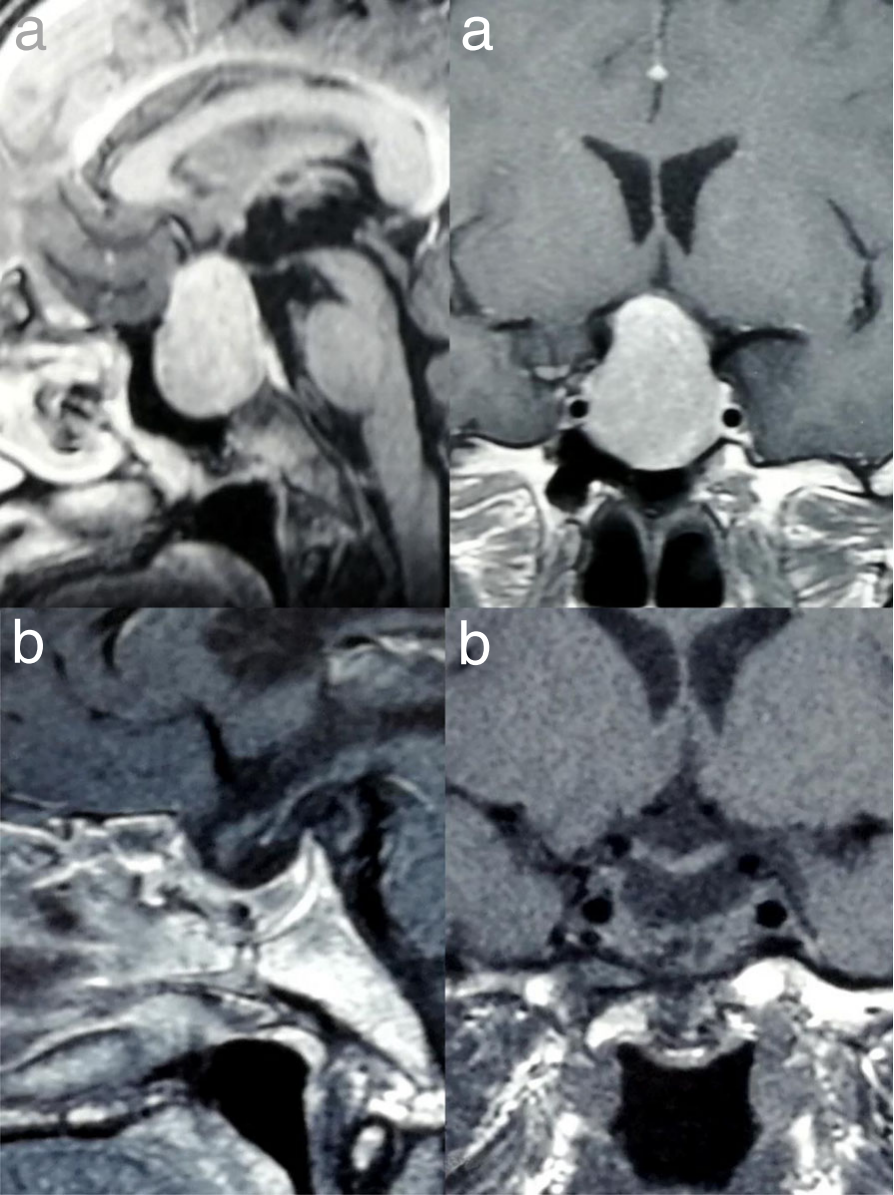
Coronal and sagittal MRI images of a patient who underwent a STT approach a. MRI image before surgery, showing a large pituitary adenoma extending suprasellar area and compressing the optic chiasm upward, b. MRI image 6 month later STT surgery showing fat grafts in the sella turcica and sphenoid sinus. The diaphragm sellae has descended into the sellar cavity and revealed complete disappearance of the tumor in the sellar and suprasellar region

This study was approved by the Neurosurgery Clinic of Pyongyang medical school hospital of the Kim Il Sung General University in DPRK. One hundred forty-five consecutive patients of pituitary adenomas treated between January 2006 and December 2013 with or/not endoscopic removal were retrospectively reviewed. All patients were treated by the same neurosurgeons. The patients, whose follow-up period was over one year, were 65 men and 81 women, with ages ranging from 20 to 65 years old (mean 46.6). Among the 116 pituitary adenomas, 39 were growth hormone-secreting, 10 were prolactin-secreting, 3 were adrenocorticotropic hormone-secreting and 61 were nonfunctioning adenomas (Table 1). All 52 functioning adenomas were microadenomas and the others were macroadenomas (Table 1, Fig. 1). Eight patients had undergone previous surgeries, including transsphenoidal surgery in five patients, craniotomy in two patients, and both in one patient.

**Table 1:** Pathology

Group	Non-functioning	GH	Prolactin	ACTH	Mixed	Etc
Control (*n* = 105)	45 (42.9)	25 (23.8)	5 (4.8)	2 (1.9)	3 (2.8)	25 (23.8)
Study (*n* = 40)	16 (40.0)	14 (35.0)	5 (12.5)	1 (2.5)		4 (10.0)

Data collected included observation of intraoperative CSF fistula, and postoperative complications including CSF fistula, meningitis and septal perforation.

### Surgical technique

The patient is placed in supine position and have been administered general anesthesia. The surgeon stands on the right side of the patient and elevates the head of the operating table by 15 degrees and tilts the face about 30 degrees to the left. The gingivobuccal sulcus, septum and face skin around the mouth was sterile and draped. Then,the gingivobuccal sulcus and nasal septum were infiltrated with lidocaine hydrochloride(HCl) and 1/200000 adrenaline. A horizontal sublabial incision was made to the depth of the periosteum of the maxilla, which was elevated superiorly to the pyriform aperture, the anterior nasal spine and the anterior angle of the nasal septum.

**Fig. 2:**
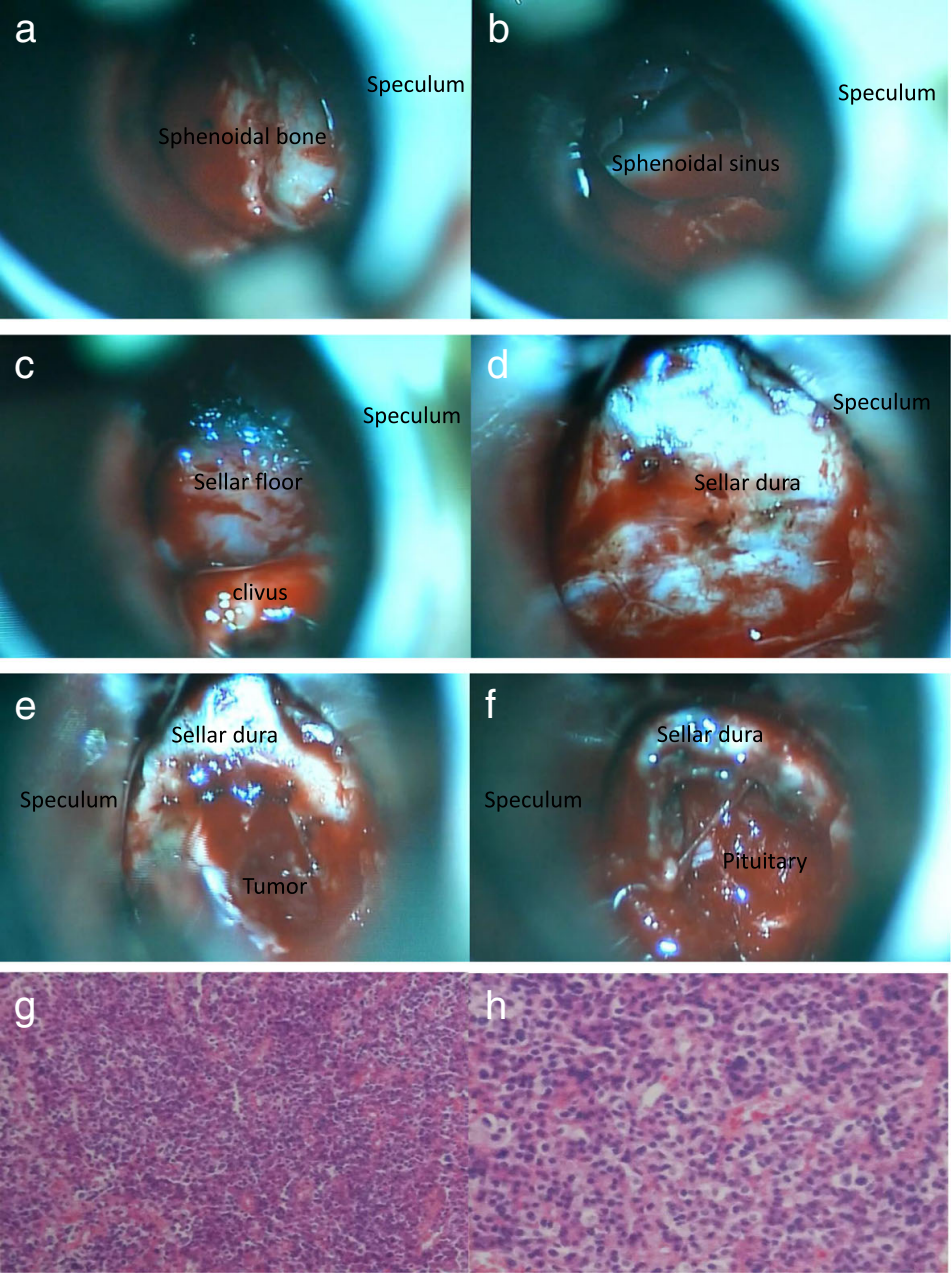
Removal of the tumor and pathological studies. **a**: Insertion of Hardy speculum to the sphenoidal bone. **b**: Confirm of ostium of sphenoidal sinus and removal of sphenoidal bone. **c**: Confirm of sellar floor. **d**: Removal of sphenoidal floor and exposure of sellar dura. **e**: The sellar dura was opened with a cruciate incision. **f**: Image after tumor removal. **g**, **h**: Pathological studies: multifunctional adenoma;PRL(+), GH(+), TSH(++)(**g**: hematoxylin and eosin staining, original magnification, *200. **h**: hematoxylin and eosin staining, original magnification, *400)

The anterior edge of the cartilaginous nasal septum was incised by a blade, and the mucous membrane was peeled from the cartilaginous and the osseous nasal septum by using a peeler so that it opened the mucosa sideways. The cartilaginous nasal septum was peeled to the right for repairment later. The Hardy speculum was inserted and the nasal mucosa of the deep part was separated from the deep osseous nasal septum. Bone nasal septum flap were reserved for restoration of sellar floor after the main procedure. Make sure that both blade of the Hardy speculum are placed symmetrically in the midline (Fig. 2a). Next, using the surgical microscope found the natural ostium of the sphenoidal sinus, and removed the sphenoidal bones with a punch(Fig. 2b). If there was an interstitial wall within the sphenoidal sinus, it will also be removed to reach the sellar floor using rongeur and a fine chisel (Fig. 2c). The dura was revealed (Fig. 2d). The test puncturing is performed with an injector of long needle to check whether or not there is a cyst or an aneurysm, and the sellar dura was dissected (Fig. 2e). The tumor was removed using suction and various types of ring curette (Fig. 2f).

Although the tumor is small, the lesion was re-examined and the residual tumor was removed under the endoscopic view. If the tumor is large and cannot be found under the microscope, using an 30 ° and 75 °endoscope searched and removed the residual tumor. The fascia that was harvested from the lateral femur was filled in the sellar space and restored to the bony nasal septum which was remained for the repairment of sellar defect [24]. The cartilaginous nasal septum was restored to the midline and the nasal mucosa was returned to its original position. Nasal cavity was filled with gauze of ointment to fix the nasal mucosa and inserted the air tube to allowed to nasal breathing. Finally, sutured gingivobuccal mucosa. The filling material was removed 3 days after surgery.

### Evaluation method

#### Pathology

In this series, pituitary adenomas were classified according to immunohistochemical staining patterns [25]. In the study group, the number of nonfunctioning(40.0%) and GH adenoma(35.0%) was large and the number of nonfunctioning adenoma(42.9%) was large in the control group (Fig. 2g, h, Table 1).

#### Size of tumor

All patients underwent contrast - enhanced computed tomography(CT) and/or magnetic resonance imaging(MRI) before surgery. The tumor size was defined as the greatest diameter of lesion on MRI. In the study group, the number of 30 to 39 mm size(42.5%) was large and the number of 20 to 29 mm(50.9%) was large in the control group (Fig. 1a, Table 2).

**Table 2:** Size of tumor

Group	~ 9	10~ 19	20~ 29	30~ 39	40~ 49	50~ 59	60~ 69	70~ 79
Control (*n* = 106)	8(7.5)	12(11.3)	54(50.9)	26(24.5)	4(3.8)	2(1.9)		
Study (*n* = 40)	1(2.5)	5(12.5)	8(20.0)	17(42.5)	5(12.5)	1(2.5)	1(2.5)	2(5.0)

#### Tumor removal

Two surgical technique, including the traditional microscopic STT approach(72.6%) and microscopic STT approach with aid of endoscope(27.4%) were utilized to remove the sellar tumors.

The extent of tumor removal was assessed by MRI after 6 months to confirm the survival of the tumor (Fig. 1b, Table 3).

**Table 3:** Tumor removal grade

Group	Total removal	Subtotal removal	Partial removal
Control(*n* = 106)	75(70.8)	16(15.1)	15(14.1)
Study(=40)	33(82.5)**	3(7.5)	4(10.0)

**(*p* < 0.01)

#### Change of visual function

We compared the preoperative and postoperative 7th day’s visual acuity and visual field tests to determine the treatment effect (Tables 4 and 5).

**Table 4:** Change of visual acuity

Group	The number of defected eye Before surgery	The number of improved eye After surgery
Control(*n* = 212)	138(100.0)	112(81.1)
Study (*n* = 80)	56(100.0)	46(82.1)

n: The number of eye

**Table 5:** Change of visual field

Group	The number of defected eye Before surgery	The number of improved eye After surgery
Control(*n* = 212)	144(100.0)	116(80.5)
Study (*n* = 80)	64(100.0)	52(81.2)

n:The numberof eye

### Statistical method

Chi Square Test and Fisher’s Exact Test was used. A *p* value< 0.05 was considered significant difference.

## Result

As shown in Table 3, in the study group, GTR was archived in 33 patients(82.5%) and in the control group was 75 patients(70.8%). There was no significant difference in visual function improvement between the two groups (Tables 4 and 5).

### Surgical outcomes

Treatment efficacy was significantly higher in the study group(97.5%) than in the control group(92.5%)(Table 6).

**Table 6:** Therapy efficiency

Group	Cure	Good	No change	Worse	Death	Efficacy (%)
Control (*n* = 106)	78(73.6)	20(18.9)	5(4.7)	1(0.9)	2(1.9)	92.5
Study (*n* = 40)	35(87.5)	4(10.0)	1(2.5)			97.5*

*(*p* < 0.05)

### Postoperative complication

As shown in Table 7, postoperative complication was recognized in the control group and there were no complications in the study group. The patients with postoperative complications in the control group were reoperation patients.

**Table 7:** Postoperative complication

Group	Rhinorrhea	Meningitis	Septal perforation
Control(*n* = 106)	1(0.94)	1(0.94)	1(0.94)
Study(=40)	0(0.0)	0(0.0)	0(0.0)

## Discussion

Since the end of the 2000s, we have begun surgery on pituitary tumors by combination of endoscopes with traditional STT approach. With aid of overseas Korean, we achieved the part of rigid endoscope, light source and air drill. Based on this, using the 0 °, 30 ° and 75° endoscope we have decided to remove residual tumor which couldnot be removed with traditional STT approach and adopted it into practice [26].

As a result of applying this method to surgery, the treatment was more effective. Although fully endoscopic endonasal surgery regards to minimally invasive method to the patients and has a higher treatment efficacy, there are still some issues to be solved in many aspects such as postoperative CSF rhinorrhea and infection. In addition, dural substitute and fibrin glue etc. used in endoscopic surgery were all imported, so it did not fit our situation [27].

Therefore, we tried to improve the tumor removal efficiency by combining endoscopes of various angles with traditional STT approach. As shown in the results of the study, the operative efficiency was high and the postoperative complications such as CSF leakage and meningitis were still few [14, 15, 28–30].

## Conclusion

STT surgery to the pituitary tumor combined with endoscope is still a significant surgical procedure. Especially postoperative complications are extremely low. It is a very economical surgical approach that allows you to achieve great success with a little cost. It is also helpful to extract a tumor that can not be seen under a microscope.

## Abbreviations

ACTH: Adrenocorticotrophic hormone
CSF: Cerebrospinal fluid
CT: Computed tomography
DPRK: Democratic people’s republic of korea
GH: Growth hormone
GTR: Gross total resection
HCl: Hydrochloride
MRI: Magnetic resonance imaging
PA: Pituitary adenoma
STT: Sublabial transseptal transsphenoidal
